# Intelligent Fuzzy System to Predict the Wisconsin Breast Cancer Dataset

**DOI:** 10.3390/ijerph20065103

**Published:** 2023-03-14

**Authors:** Yamid Fabián Hernández-Julio, Leonardo Antonio Díaz-Pertuz, Martha Janeth Prieto-Guevara, Mauricio Andrés Barrios-Barrios, Wilson Nieto-Bernal

**Affiliations:** 1 Faculty of Economics, Administrative and Accounting Sciences, Universidad del Sinú Elías Bechara Zainúm, Montería 230002, Colombia; 2 Departamento de Ciencias Acuícolas–Medicina Veterinaria y Zootecnia (CINPIC), Universidad de Córdoba, Montería 230002, Colombia; 3 Systems Engineering Department, Universidad de la Costa, Barranquilla 080001, Colombia; 4 Facultad de Ingeniería, Departamento de Ingeniería de Sistemas, Universidad del Norte, Barranquilla 80001, Colombia

**Keywords:** fuzzy system, breast cancer, clusters, pivot tables

## Abstract

Decision Support Systems (DSSs) are solutions that serve decision-makers in their decision-making process. For the development of these intelligent systems, two primary components are needed: the knowledge database and the knowledge rule base. The objective of this research work was to implement and validate diverse clinical decision support systems supported by Mamdani-type fuzzy set theory using clustering and dynamic tables. The outcomes were evaluated with other works obtained from the literature to validate the suggested fuzzy systems for categorizing the Wisconsin breast cancer dataset. The fuzzy Inference Systems worked with different input features, according to the studies obtained from the literature. The outcomes confirm that most performance’ metrics in several cases were greater than the achieved results from the literature for the output variable for the different Fuzzy Inference Systems—FIS, demonstrating superior precision.

## 1. Introduction

Cancer is a group of diseases that cause cells in the body to change and spread out of control [1]. Breast cancer is considered the second most common cancer among women in the United States (some kinds of skin cancer are the most common). According to [2], among the signs and symptoms of breast cancer, we can find a lump or swelling in the breast, upper chest, or armpit; changes in the size or shape of the breast; a change in skin texture and color; rash, crusting, or modifications to the nipple. For the mentioned causes, it is critical to create simulations that help in the decision-making process for initial detection, proper therapy, and therapy [3] to achieve a rapid diagnosis. Fuzzy systems have been used for breast cancer classification [4,5], among other uses. Fuzzy set theory is known as the basis of all fuzzy logic methods [6]. Fuzzy set theory was proposed by Zadeh [7] as an extension of the classical set theory to model sets, whose elements have degrees of membership [8]. According to [7], a fuzzy set is a class of objects with a continuum of grades of membership. Such a set is characterized by a membership (characteristic) function which assigns each object a category of membership ranging between zero and one. A degree of one means that an object is a member of the set, a value of zero means it is not a member, and a value somewhere in-between shows a partial degree of membership [8]. This partial degree of membership is also known as the membership function. The notions of inclusion, union, intersection, complement, relation, convexity, etc., are extended to such sets, and various properties of these notions in the context of fuzzy sets are established [7].

The fuzzy set theory provides the tools to effectively represent linguistic concepts, variables, and rules, becoming a natural model to represent human expert knowledge [9]. According to [8], a linguistic value refers to a label for describing the experience that has meaning determined by its degree of the membership function. One of the most fruitful developments of fuzzy set theory is Fuzzy Rule-Base Systems—FRBs [8]. The Fuzzy Decision Support System (FDSS) was developed to convert knowledge from experts based on fuzzy rules to improve decision making [6]. For the development of this kind of Decision Support system, the Mamdani-type FIS is widely used [10,11]. Fuzzy Decision Support Systems are used in the knowledge field of Medicine [11,12,13,14,15].

For these reasons, the main goal of this research work was to create different intelligent fuzzy systems using clusters and dynamic tables for the classification of the Wisconsin breast cancer dataset. To validate the proposed models, the fuzzy inference systems—FIS—were conceived to classify the mentioned dataset and contrasted with other artificial intelligent technique models obtained from the literature. The originality of this work lies in its generation of membership functions. Some authors use different approaches for generation. We can find 2N + 1 regions, FCM, neural networks, GAs, etc. In our case, we proposed using clustering methods for this step. The main difference at this stage is that no fixed or random membership functions were generated, such as those caused by those works that used classical methods or were based on evolutionary algorithms, neural networks, or swarm intelligence techniques. Another difference between this study and the related works using neural networks, evolutionary or swarm algorithms is that we did not use random numbers, or any chromosome or particle scheme. Regarding the generation of the rule base for the system, some authors also used the same previously mentioned methods. The main difference with our work is that our approach uses pivot tables instead of other techniques. Other authors initialize with random weights and bias for each hidden neuron (neural networks), adjusting them through optimization functions such as gradient descendent and non-linear activation functions. Other methods use random schemes to generate the fuzzy rules, using the objectives function to adjust membership functions and the rule base, i.e., MSE. Our study did not propose using any objective function as a minimization problem. In addition, our study did not offer to employ or calculate any distances, attractiveness, or another parameter to generate the fuzzy rule base. The only component used for this task was pivot tables. Pivot tables did not use any calculation method or random or manual parameters (only sorting options). The main job of this technique is to eliminate redundant information.

## 2. Material and Methods

**Figure 1:**
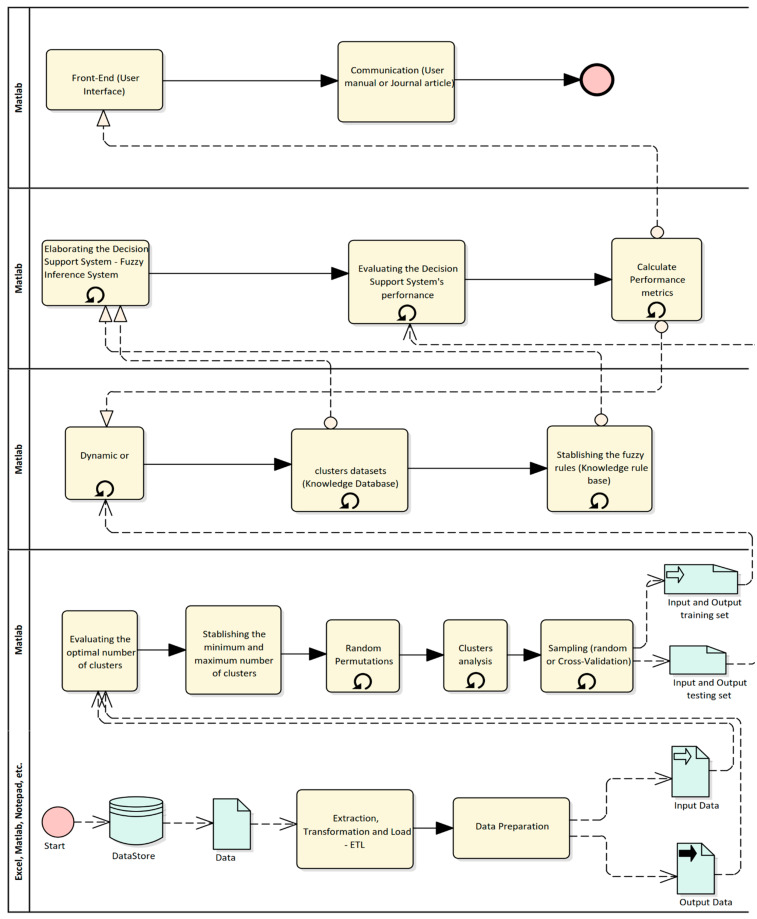
The proposed five layers architecture framework. Source: Own elaboration.

To validate the framework proposed by [16,17]—(Figure 1), a case study was designed and implemented. Each of the stages suggested in the framework will be explained.

### 2.1. Identifying the Dataset

**Figure 2:**
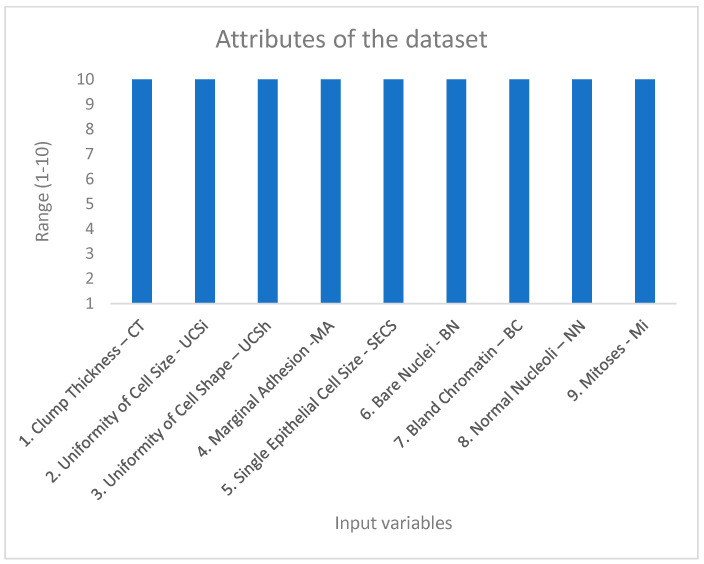
Input variables of the Wisconsin Breast Cancer dataset.

**Figure 3:**
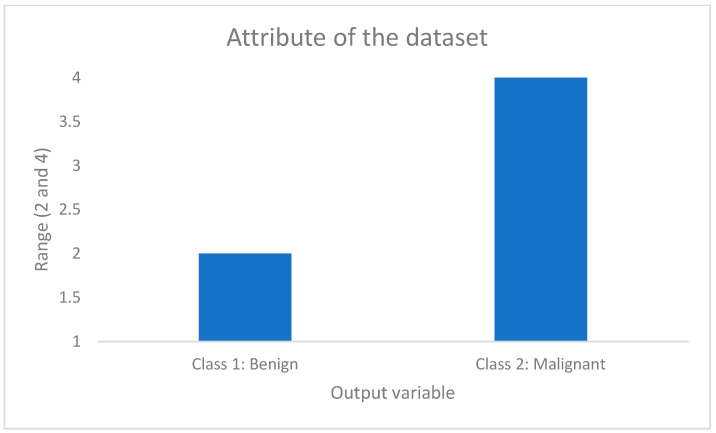
Output variable of the Wisconsin Breast Cancer dataset.

The dataset used for this research was obtained from the UCI Machine Learning repository to evaluate the efficacy of the proposed framework using the Wisconsin Breast Cancer Dataset (WBCD) [18,19]. The dataset was compiled from the patients of the University of Wisconsin–Madison Hospitals. The instance of this dataset is a 699 data pair. The dataset contains missing values. In this case, the character “?” was changed to zero. This change was made because the values of the input variables are within the range of 1 to 10. In this case, the symbol “?” represents a lost value. It was decided not to apply statistical methods such as the mean of series, mean or median of nearby points, or linear interpolation because these variables are discrete variables. The result of the application of these methods is a continuous variable. When applying the mentioned methods, the results were always the same: 3.5. This makes the decision-making process difficult because it is not known to what value to assign it if it is three or four. In this case, the operation was manual, replacing the values of the symbols with the number zero. The number zero indicates that you do not have the value of that variable. The missing values belong to the variable Bare Nuclei (BN). Other changes were made in the dataset to adjust the number of the classification: two to one for benign instances and four to two for malignant instances. This change was made because if the system worked with these two values (2 and 4), some of the outputs obtained by the fuzzy inference system could be in the middle of the range of these two values; that is, number three could be obtained as an answer. In that case, this value could hinder the decision-making process, because this value cannot tell us if the selected instance is malignant or benign. This process was carried out manually, replacing the values of the output variable: two by one and four by two, comprising 458 benign cases and 241 malign cases. In this case, according to the data, the two classes are imbalanced. In this case, when an unbalanced dataset is reached, we usually obtain a high precision value in the Majority class (benign cases) and a low recall in the Minority class (malignant cases). However, according to the results obtained by the fuzzy inference system with better results (Table 1 and Table 2), these were excellent because the specificity value was 1.0, indicating that 100% accuracy was obtained in the minority class (malignant instances). Because of this situation, the research team had no need to use strategies for handling unbalanced data, such as model parameter adjustments, modifying the dataset, using artificial or synthetic samples, or using balanced ensemble methods. The descriptive statistics of the dataset can be found in Onan [20]. There are nine input features and one output feature (Figure 2 and Figure 3).

The attributes of the dataset are:

**Table 1:** Confusion matrix for WCDB dataset.

		Specialists	
		Benign	Malign
DDFDSS	Benign	**455**	3
	Malign	0	**241**

DDFDSS: Data-driven fuzzy decision-support system. Bold values represent accurate forecasts.

**Table 2:** Performance metrics obtained with the proposed framework.

[16]	CV			RS		
Num of variables: [2 4 5 6 8]	K-Means	Ward	FCM	K-Means *	Ward	FCM *
Num of Rules or Hidden neurons /technique	248	233	190	207	208	168
Accuracy (%):	99.3%	99.4%	99.1%	99.0%	99.57%	98.43%
Sensitivity:	0.9857	0.9853	0.9851	0.9916	0.9877	0.9637
Specificity:	0.9969	0.998	0.9939	0.9892	1.0000	0.9956
F-Measure:	0.9899	0.9907	0.9868	0.9854	0.9938	0.9775
Area under curve:	0.9933	0.9942	0.9903	0.9874	0.9967	0.986
Kappa statistics:	0.9845	0.9858	0.9798	0.9778	0.9905	0.9654

CV: Cross-validation method. RS: random sampling. * Significant difference at 95% of the Confidence Interval between them.

### 2.2. Data Preparation (Crisp Inputs)

The first activity was to identify inputs and outputs features. In the experiment, all input and output features were chosen. In this phase, the data were processed because the dataset contains missing values. The symbol “?” was changed for zero (as mentioned above). In this stage, the preprocessed technique applied was clustering. This method is explained in Section 2.7 [21].

### 2.3. Reviewing Existing Models

In this phase, a search of the different related works about the problem was carried out. Several indexed databases, such as Scopus, Science Direct, among others, were used. The outcome of this stage is revealed in the discussion segment.

### 2.4. Evaluating the Optimal Number of Clusters

In this stage, pivot tables were applied to determine the total number of rows for every input and output feature. For the experiment, the optimum number of clusters was 10.

### 2.5. Setting a Number of Clusters (Minimum and Maximum) According to the Previous Evaluation

The minimum used value was two; the maximum quantity of clusters was ten for all input features. Two clusters were used for the output feature. The clusters’ optimal number was stablished as the maximum number of clusters to avoid greater fuzzy sets numbers in the output (input variables interaction).

### 2.6. Random Permutations

For the dataset, the observed inputs and outputs values were randomized and commuted when used the suggested algorithms.

### 2.7. Cluster Analysis (Fuzzification Process)

In this phase, three types of clusters (kmeans, Ward, and Fuzzy C-Means) were achieved and analyzed using the range of solutions created in the preceding stage. For the first two clustering algorithms, the Euclidean distance was selected as the default. For the fuzzy c-means clustering algorithm, the default value for the exponent for the fuzzy partition matrix U was 50, the maximum number of iterations was 100, the minimum improvement in the objective function was 1 × 10^−5^, and the objective function was displayed as false (zero). The selection criteria for clustering algorithm must correspond to the knowledge of the topic.

The maximum number of clusters for each input and output feature for the dataset was the values of the optimum cluster (ten for the inputs variables and two for the output variable).

### 2.8. Sampling Datasets (Cross-Validation or Random Sampling)

For the experiments, two methods of random data sampling were used. The first method used was random sampling, and the other one was the cross-validation method. For the random sampling, the user could select the percentage for every subset (training, validation and test), and the number of iterations. The default values for this kind of data partition method were as follows: training dataset: 70%; validation dataset: 30%; test dataset: 0% [22]; and number of iterations: 3000. For the cross-validation process, the k-fold method was selected. For the case study, the used value of k by default was 10. This value was selected because it is one of the most used in this type of validation method of machine learning models. In this case, the dataset was divided into 10 equal parts, with an equal number of training and validations. For all the experiments, we used a personal computer (PC). The computer’s specification for the algorithm’s implementation was an AMD A12-9720P Radeon R7, 12 compute cores 4C + 8G 2.70 GHz with 16.00 GB RAM, Hard Disk Drive (HDD) of 1 Tera Byte (TB).

### 2.9. Pivot Tables

For the experiment, the unique tables command was applied for the implementation of the subsequent sub-stages.

#### 2.9.1. Combining Different Input Variable Clusters Datasets

This phase comprises creating arrangements between input features and the sets of output features using dynamic tables. The permutations were carried out by applying the command “nchoosek” and “unique” for matrixes. The first command sends back a matrix comprising all possible permutations of the elements of vector v taken k at a time. The second command returns a copy of dataset A, which contains only the sorted unique observations [23].

#### 2.9.2. Stablishing the Fuzzy Rules

This phase is established on the preceding one. The procedures carried out with the use of the dynamic tables one or several permutations can be used to make the rule bases for the FIS. To achieve this, we can use the unique command to avoid rules duplication. For all the experiments, the Center of Gravity method was chosen as the de-fuzzification process by default, contemplating all output options and converting the fuzzy set originated by inference into a numerical value, as proposed by [24,25]. Generally, software programs for the implementation of this type of model use the Centroid method for defuzzification. This method can be considered a weighted average, where the weights are represented by μA (xi), which indicates the degree of membership of the value xi with the concept modeled by the fuzzy output set A, and which, in its compound shape, is calculated by:

(1) $$Z=\frac{µc\left(z\right)z\delta z}{µc\left(z\right)\delta z}$$

where *Z* is the consequent variable and µ*_c_(z)* is the function of the composed shape. The result of the defuzzification process *Z* can be continuous or discrete [26].

### 2.10. Elaborating the Decision Support System Based on Fuzzy Set Theory (Inference Engine)

For the experiments, the FIS’ implementation was carried out in the MATLAB^®^ R2017a software. In this stage, the aim is to join all components cited above in order. The first step was to generate a new FIS file. We put a name to the created FIS file. To define the defuzzification process, by default, we selected the centroid defuzzification method (a choice between “Centroid”, “som—small of maximum”, “mom—mean of maximum”, or “lom—large of maximum”). For all the experiments’ implementation, the fuzzy logic toolbox was not used because this tool does not work with a data-driven approach. This means that it is not an automated fuzzy inference systems developer. All fuzzy inference systems designed with this toolbox are developed manually. Instead, we used our algorithms (Pseudocodes available in the appendixes of Reference [16]).

### 2.11. Evaluating the Fuzzy System Performance (Defuzzification and Crisp Outputs)

For the experiments, the system’s performance was measured through some of the following metrics: the classification accuracy (ACC), sensitivity, specificity, function of measure, area under the curve, and Kappa statistics. Additionally, we performed a statistical significance test called McNemar’s test. The aim is to examine whether the differences between the prediction performances of feature subsets are statistically significant or not [27]. This test was applied in those results that used random sampling as a data partition method only, because using a cross-validation, we have more than one confusion matrix; we have k-folds confusion matrixes; however, we calculate the test for all three clustering methods in all results.

## 3. Results and Discussion

The following were the obtained outcomes for the cited dataset:

The confusion matrix for the mentioned data-driven fuzzy clinical decision support system (DDFCDSS) are shown in Table 1. The performance metrics obtained with our proposed framework are shown in Table 2. The best results for a set of five features were obtained via the Ward clustering method.

As can be seen, the DDFCDS had a specificity value of 100%, suggesting an outstanding performance predicting or classifying the true negatives cases of the WBCD. It means that all malignant cases were classified correctly. According to the confusion matrix, there are only three true positive values that are misclassified corresponding to a sensitivity value of 0.9877.

In the following pages, we are going to compare the results obtained from the literature with our results. The results shown in the tables below correspond to the same characteristics noted by the researchers using the same dataset (WBCD). We used the same data partition method, the same features.

According to the results, for the WBCD, the greatest performance belongs to Onan [20]. The author used a classification model based on the fuzzy–rough nearest neighbor algorithm, consistency-based feature selection, and fuzzy–rough instance selection for a medical diagnosis. He used a 10-fold cross-validation method as a data partition method. As can be seen in Table 3, the classification accuracy for his results was 99.72%, and the maximum value for classification accuracy of our results belongs to the k-means 10-fold cross-validation method. The sensitivity value for the author was 100%; however, his specificity value was 0.9947. Our results show the opposite. Our specificity value was 1.0, and the sensitivity value was 0.9703. The performance metric sensitivity indicates the true positive (TP) rate, and specificity means the true negative (TN) rate [28]. According to [28], in breast cancer, the TP signifies cases that are correctly categorized in the benign tumor, and the TN characterizes cases that are correctly categorized in the malignant tumor. This result shows that our model predicts 100% of the true negative values. In this case, we can state that if a tumor is malignant, the fuzzy inference system is going to be classified as malignant with 100% accuracy.

Through the comparison of the three clustering methods results, we found that McNemar’s test indicated that none of them perform significantly better than the others, indicating that all the DDFCDSS have the same classification error rates. The test results were Ward vs. k-means: $X_{1}^{2}$ = 0.0455; k-means vs. FCM: $X_{1}^{2}$ = 0.0, and Ward vs. FCM: $X_{1}^{2}$ = 0.12903, respectively.

Ref. [29] proposed a Breast Cancer Computer Aid Diagnosis (BC-CAD) based on joint variable selection and a Constructive Deep Neural Network “ConstDeepNet”. A feature variable selection method was applied to decrease the number of inputs used to train a Deep Learning Neural Network. The authors used five-fold cross-validation as a partition data method. The classification accuracy for the set of features mentioned in Table 4 is 96.2%. Our results were higher than those obtained for these authors. Our classification accuracy using the cross-validation data partition method with k = 5 was 98.37%. For comparison of the three clustering methods, the McNemar’s test results are as follows: K-means vs. Ward: $X_{1}^{2}$ = 0.3636; k-means vs. FCM: $X_{1}^{2}$ = 1.8947, and Ward vs. FCM: $X_{1}^{2}$ = 0.5625, indicating no significant differences between them. For the case of the second set of features used by the authors (Table 5), the classification accuracy obtained by the constructive deep neural network was 96.6%. Our results for the same set of features were higher than those obtained by the authors. Regarding the McNemar’s test results for the three clustering methods, they indicate that there is no significant difference among them. The test values are k-means vs. Ward: $X_{1}^{2}$ = 0.3636; k-means vs. FCM: $X_{1}^{2}$ = 1.8947; Ward vs. FCM: $X_{1}^{2}$ = 0.5625.

Another author who works with the same dataset was [30]. The authors introduced an automated medical data classification method using wavelet transformation (WT) and interval type-2 fuzzy logic system (IT2FLS. The authors used five-fold cross-validation as a data partition method. The classification accuracy for this set of features was 97.88% (Table 6). The best performance of the three clustering methods was obtained for the Ward method, with 96.68% showing a better performance between the models. Regarding the McNemar’s test results, the values were: K-means vs. Ward: $X_{1}^{2}$ = 14.0192; K-means vs. FCM: $X_{1}^{2}$ = 0.0294; Ward vs. FCM: $X_{1}^{2}$ = 12.5000. The values higher than 3.84 can be interpreted as a significant difference. This means that we reject the null hypothesis and accept the alternative hypothesis indicating that the algorithms do not have the same classification error rate. In this case, the DDFCDSS using the k-means and FCM have the same classification error rates.

Ref. [31] developed a manually Mamdani-type fuzzy inference system (FIS). The authors proposed a framework for the development of fuzzy inference systems using dynamic tables and clusters; however, the framework does not support a data-driven approach. The classification accuracy for the authors was 98.58% (Table 7), showing a sensitivity of 100%; however, the specificity is lower than our results. The best performance for our DDFCDS was obtained by the k-means method using random sampling as a data partition method. McNemar’s test indicates that k-means vs. FCM has significant difference between them. The test results values are the following: k-means vs. Ward: $X_{1}^{2}$ = 3.0625; k-means vs. FCM = $X_{1}^{2}$ = 8.6538, and Ward vs. FCM: $X_{1}^{2}$ = 2.2273.

The other authors who had better results than our DDFCDSS were Abdel-Zaher and Eldeib [32]. Ref. [32] proposed an integration between Wavelet Transformation (WT) and Interval Type-2 Fuzzy Logic Systems (IT2FLS) to cope with both high-dimensional data challenge and uncertainty. The authors used all input variables and used random sampling (70–30%) for data partition. The classification accuracy for this author was 99.68%, with a sensitivity of 100% and a specificity of 0.9947 (Table 8). Our best performance using the same data partition was the k-means DDFCDSS, with a classification accuracy of 98.86. McNemar’s test showed that the highest performance of the DBN model, which uses nine variables, was significantly better than our Data-Driven Fuzzy CDSS, which has the highest performance. For the comparison among the three clustering methods, the test results suggest that they have no significant difference among them. The values for the test are K-means vs. FCM: $X_{1}^{2}$ = 2.400; K-means vs. Ward: $X_{1}^{2}$ = 1.250; Ward vs. FCM: $X_{1}^{2}$ = 0.

Ref. [28] proposed a fully connected layer first CNN (FCLF-CNN), in which the fully connected layers are embedded before the first convolutional layer. The authors used two data partition methods for the experiments. The authors used a five-fold cross-validation approach. The obtained results for this scheme are presented in Table 8. The authors also used two settings for the random sampling (train: 50%, test: 50%, and train: 75%, test: 25%). The results obtained from the random sampling were 98.57% and 98.86%, respectively. As can be seen in Table 8, our proposed framework could obtain a better performance in the cross-validation method: The Ward method obtained a classification accuracy of 98.84%. Regarding the random sampling method, the k-means obtained the best performance with a classification accuracy of 98.86%, which was similar to the results obtained by [28] for the same dataset and random sampling configuration.

The main differences and similarities between the mentioned related works with the proposed framework are as follows:

(1) Like all the mentioned works, we identified all the input and output variables for the Wisconsin Breast cancer dataset classification problem, including the related works using the same datasets.
(2) To generate the membership functions, the mentioned authors used different approaches, including logistic regression, support vector machine, random forest, fuzzy c-means, neural networks (MLP, DNN), K-nearest neighbor, genetic algorithms, etc. In our case, we proposed using clustering methods for this step. Among the clustering methods, we used k-means, the Ward method, and FCM. The main difference at this stage is that no fixed or random membership functions, such as those caused by those works that used classical methods or were based on evolutionary algorithms (GAs, FA, BBO), neural networks, or swarm intelligence techniques (PSO, ACO), were generated. Instead, the users can select the number of membership functions (number of clusters) they want to use for each input/output variable for each classification problem. Another difference between our framework and the related works using neural networks, evolutionary, or swarm algorithms is that we did not use random numbers, chromosomes, or particle schemes. Instead, our membership functions were obtained using well-known and recognized clustering methods. They indicate whether a sample belongs to a group, obtaining a vector with the values of one of the groups assigned to the input/output variable. Thus, the assignation of the number of groups is not random. In addition, we did not use any random population, random particles, random weights, or bias.
(3) To generate the system’s rule base, the main difference between our work and the mentioned works is that our approach uses pivot tables instead of other techniques. As mentioned, every method for generating the intelligent systems’ rules or connections has its own characteristics. Some initialize with random weights and bias for each hidden neuron (neural networks), adjusting them through optimization functions such as gradient descendent and non-linear activation functions. Other methods use random schemes to generate the fuzzy rules, using the objectives function to change membership functions and the rule base, i.e., Mean Square Error (MSE). Our proposed framework did not offer the chance to use any objective function as a minimization problem. Additionally, our framework did not suggest using or calculating any distances, attractiveness, or parameter to generate the fuzzy rule base. The only component used for this task was pivot tables. Pivot tables did not use any calculation method or random or manual parameters (only sort options). The primary mission of this technique is to eliminate redundant information.

Our framework’s main advantage is our algorithms’ simplicity using only primitive mathematical operators and clustering operations (Appendixes shown in Reference [16]). Our framework’s parameters are as follows: (a) To select inputs and outputs variables. (b) To choose the clustering algorithm (k-means, Ward, FCM). (c) To select the number of Membership Functions—MFs (number of clusters)—that the user wants. (d) To adopt the data partition method (random sampling or cross-validation). (e) To select the number of features the user wants to use (feature extraction). (f) To set the parameters according to the selected data partition method. For example, if the user selects random sampling, they must determine the percentages for training, validation, and test datasets, and the number of iterations. Otherwise, the users must choose the cross-validation partition method (‘k’,’KFold’, ‘Holdout’,’LeaveOut’, or ‘Resubstitution’) and the iterations’ number.

As can be read, among the parameters, there is nothing about a lower–upper bound, any random number, any inertia, momentum, distance, weight, bias, or population size to calculate or initialize. This means that the result of each iteration for every combination (Section 2.9.1. Combining different cluster datasets) is a fuzzy inference system because it is not necessary to adjust or optimize weights, bias, or any objective or fitness function.

It should be noted that the only parameters configured internally were those used for the clustering methods, and they are mentioned in Section 2.7, Clusters analysis (Fuzzification process). These criteria have low computational requirements, offering precision, processing speed, and interpretability of the rules.

## 4. Conclusions

The main objective of this research work was to implement and validate different decision support systems founded on Mamdani-type fuzzy set theory using clusters and dynamic tables. As could be demonstrated, in some cases, the proposed fuzzy models showed the best-performing indices related to this dataset, surpassing the outcomes obtained from advanced techniques (deep learning) such as Deep Neural Network and Convolutional Neural Networks. The obtained outcomes for the used performance metrics were nearer to one, indicating a robust fit between the predicted and the observed data. The area under the curve for this dataset ranged between 0.90 and 1.0, representing an excellent classification task [34]. The selected features shown in Table 2 for both data partition methods were: Uniformity of Cell Size (UCSi), Marginal Adhesion (MA), Single Epithelial Cell Size (SECS), Bare Nuclei (BN), and Normal Nucleoli (NN), indicating that it is not necessary to carry out the mitosis process accelerating diagnosis and a possible treatment [16,31]. According to the McNemar’s test results for the three clustering methods, the k-means have significant difference at 95% of the confidence interval with the FCM clusters method ($X_{1}^{2}$ = 5.7857), indicating that these two clusters methods have different error rate. For the other two clusters methods, the test evidenced that the clustering methods did not perform significantly differently.

We can conclude that the current framework provides a real pattern for the development of data-driven Mamdani-type fuzzy decision-support systems for classification problems. Another conclusion is the computational performance of the algorithms has homogeneous behavior when running with similar datasets.

Other main future work aims to implement this in other software development platform such as python, Scilab, and Octave, among others.

## Figures and Tables

**Table 3:** Performance metrics obtained with our proposed framework compared with results obtained by [20].

	Onan [20]	CV			RS		
Number of Variables	[1 2 4 5 6 7 8]	K-Means	Ward	FCM	K-Means ^NS^	Ward ^NS^	FCM ^NS^
Num of Rules or Hidden neurons/technique	FRNN	312	338	260	253	249	244
Accuracy (%):	99.72%	98.94%	98.53%	98.66%	98.00%	98.28%	96.71%
Sensitivity:	1.0000	0.9703	0.9594	0.9694	0.9451	0.9526	0.9258
Specificity:	0.9947	1.0000	1.0000	0.9960	1.0000	1.0000	0.9910
F-Measure:	0.9970	0.9849	0.9792	0.9808	0.9718	0.9757	0.9537
Area under curve:	1.0000	0.9919	0.9888	0.9880	0.9737	0.9869	0.9710
Kappa statistics:	0.9943	0.9768	0.9679	0.9704	0.9563	0.9624	0.9282

CV: cross-validation. RS: random sampling. FRNN: fuzzy–rough nearest neighbor. ^NS^: Non-significant difference at 95% of the confidence interval.

**Table 4:** Performance metrics obtained with our proposed framework compared with results obtained by [29].

	Zemouri, Omri, Devalland, Arnould, Morello, Zerhouni and Fnaiech [29]	CV			RS		
Number of Variables	[1 4 5 6 8 9]	K-Means	Ward	FCM	K-Means ^NS^	Ward ^NS^	FCM ^NS^
Num of Rules or Hidden neurons /technique	DNN	198	214	199	192	193	180
Accuracy (%):	96.2%	98.37%	98.26%	98.31%	99.00%	98.86%	98.86%
Sensitivity:	-	0.9713	0.9736	0.9621	0.9875	0.9915	0.9794
Specificity:	-	0.9903	0.9873	0.9947	0.9913	0.9870	0.9934
F-Measure:	-	0.9765	0.9747	0.9759	0.9854	0.9832	0.9835
Area under curve:	-	0.9832	0.981	0.9848	0.9884	0.9844	0.9883
Kappa statistics:	-	96.40%	96.14%	96.29%	97.8%	97.45%	97.47%

CV: cross-validation. RS: random sampling.—Not mentioned in the literature. DNN: Deep Neural Network. ^NS^ Non-significant difference at 95% of the confidence interval.

**Table 5:** Performance metrics obtained with our proposed framework compared with results obtained by Zemouri, Omri, Devalland, Arnould, Morello, Zerhouni, and Fnaiech [29].

	Zemouri, Omri, Devalland, Arnould, Morello, Zerhouni, and Fnaiech [29]	CV			RS		
Variables	[1 2 5 6 7 8]	K-Means	Ward	FCM	K-Means ^NS^	Ward ^NS^	FCM ^NS^
Num of Rules or Hidden neurons/technique	DNN	212	221	147	183	198	178
Accuracy (%):	96.60%	98.63%	98.51%	96.25%	99.00%	98.86%	98.00%
Sensitivity:	-	0.9684	0.9684	0.959	0.9875	0.9794	0.9595
Specificity:	-	0.996	0.9943	0.9646	0.9913	0.9934	0.9912
F-Measure:	-	0.9803	0.9787	0.9448	0.9854	0.9835	0.9713
Area under curve:	-	0.9878	0.9861	0.9552	0.9884	0.9883	0.9808
Kappa statistics:	-	96.98%	96.73%	91.65%	97.8%	97.5%	95.6%

CV: cross-validation. RS: random sampling.—Not mentioned in the literature. DNN: deep neural network. ^NS^: No significance difference at 95% of confidence interval.

**Table 6:** Performance metrics obtained with our proposed framework compared with results obtained by Nguyen, Khosravi, Creighton, and Nahavandi [30].

	Nguyen, Khosravi, Creighton, and Nahavandi [30]	CV			RS		
Num of Variables	[3 4 5]	K-Means	Ward	FCM	K-Means ^1^	Ward ^1,2^	FCM ^2^
Num of Rules or Hidden neurons /technique	WT—IT2FLS	184	185	185	46	50	51
Accuracy (%):	97.88%	96.68%	96.54%	96.54%	97.14%	97.34%	96.73%
Sensitivity:	0.9850	0.9691	0.9642	0.9642	0.9784	1.0000	1.0000
Specificity:	0.9650	0.9657	0.9661	0.9661	0.9679	0.9598	0.9532
F-Measure:	-	0.9510	0.9490	0.9490	0.9576	0.9623	0.9484
Area under curve:	0.9750	0.9589	0.9580	0.9580	0.9634	0.9571	0.9437
Kappa statistics:	-	0.9259	0.9228	0.9228	93.6%	56.6%	55.3%

CV: Cross-validation. RS: random sampling.—Not mentioned in the literature. WT: Wavelet transformation. IT2FLS: interval type-2 fuzzy logic system. ^1^ A significant difference between them. ^2^ significant differences between them at 95% of the Confidence Interval.

**Table 7:** Performance metrics obtained with our proposed framework compared with results obtained by [31].

	[31]	CV			RS		
Num of Variables	[1 2 6]	K-Means	Ward	FCM	K-Means *	Ward	FCM *
Num of Rules or Hidden neurons/technique	FIS 39	42	52	43	50	48	41
Accuracy (%):	98.58%	93.39%	90.80%	76.98%	94.56%	90.84%	93.99%
Sensitivity:	1.0000	0.9956	0.9951	0.9885	0.9677	0.9683	0.9628
Specificity:	0.5000	0.9099	0.8783	0.7420	0.9357	0.8863	0.9298
F-Measure:	0.9928	0.8943	0.8465	0.4953	0.9170	0.8512	0.9079
Area under curve:	-	0.9050	0.8675	0.6672	0.9437	0.8731	0.9207
Kappa statistics:	96.8%	84.7%	78.3%	39.4%	87.7%	78.6%	86.35%

CV: Cross-validation. RS: random sampling.—Not mentioned in the literature. FIS: Fuzzy inference system. * Significant difference between the methods at 95% of the Confidence Interval.

**Table 8:** Performance metrics obtained with our proposed framework compared with results obtained by other authors obtained from the literature.

	[32]	[33]	[28]	CV			RS		
Num of Variables	[1 2 3 4 5 6 7 8 9]			K-Means	Ward	FCM	K-Means ^NS^	Ward ^NS^	FCM ^NS^
Num of Rules or Hidden neurons /technique	DBN -4-2	SMO	FCLF-CNN	343	309	306	195	251	217
Accuracy (%):	99.68%	72.70%	98.71%	98.50%	98.84%	98.74%	98.86%	98.28%	98.43%
Sensitivity:	1.000	-	0.976	0.9584	0.971	0.9649	0.9755	0.9562	0.9563
Specificity:	0.9947	-	0.9943	1.000	0.998	1.000	0.9956	0.9978	1.0000
F-Measure:	-	0.71	-	0.9787	0.9835	0.9821	0.9835	0.9756	0.9777
Area under curve:	-	0.63	0.9816	0.9885	0.9903	0.9904	0.9893	0.9859	0.9880
Kappa statistics:	-	-	-	0.9671	0.9745	0.9724	97.48%	96.24%	96.55%

CV: Cross-validation. RS: random sampling.—Not mentioned in the literature. DBN: deep belief network. SMO: Sequential minimal optimization. FCLF: Fully connected layer first. CNN: Convolutional neural network. ^NS^: Not significant difference at 95% of the confidence interval.

## Data Availability

The data supporting this study are available at https://archive.ics.uci.edu/ml/datasets/breast+cancer+wisconsin+%28original%29 (accessed on 1 November 2021).
